# A multiobjective multi-view cluster ensemble technique: Application in patient subclassification

**DOI:** 10.1371/journal.pone.0216904

**Published:** 2019-05-23

**Authors:** Sayantan Mitra, Sriparna Saha

**Affiliations:** Department of Computer Science and Engineering, Indian Institute of Technology Patna, India; Northeast Electric Power University, CHINA

## Abstract

Recent high throughput omics technology has been used to assemble large biomedical omics datasets. Clustering of single omics data has proven invaluable in biomedical research. For the task of patient sub-classification, all the available omics data should be utilized combinedly rather than treating them individually. Clustering of multi-omics datasets has the potential to reveal deep insights. Here, we propose a late integration based multiobjective multi-view clustering algorithm which uses a special perturbation operator. Initially, a large number of diverse clustering solutions (called base partitionings) are generated for each omic dataset using four clustering algorithms, viz., k means, complete linkage, spectral and fast search clustering. These base partitionings of multi-omic datasets are suitably combined using a special perturbation operator. The perturbation operator uses an ensemble technique to generate new solutions from the base partitionings. The optimal combination of multiple partitioning solutions across different views is determined after optimizing the objective functions, namely *conn*-XB, for checking the quality of partitionings for different views, and agreement index, for checking agreement between the views. The search capability of a multiobjective simulated annealing approach, namely AMOSA is used for this purpose. Lastly, the non-dominated solutions of the different views are combined based on similarity to generate a single set of non-dominated solutions. The proposed algorithm is evaluated on 13 multi-view cancer datasets. An elaborated comparative study with several baseline methods and five state-of-the-art models is performed to show the effectiveness of the algorithm.

## Introduction

In the field of biology and medicine, classification has wide range of applications [1]. With the advancement in microarray technology, generation of thousands of gene sequence data points for cancer-tissue datasets has become possible. It is possible to accurately differentiate between different categories of cancers by analyzing the gene expression values of cancer tissues over different conditions or time points. Classification of patients into subgroups can improve the diagnostic and treatment. Available methods for patient stratification are dependent on gene sequence data and patients are grouped based on the expression profiles [2, 3]. In addition to gene sequence data other data types, like miRNA (microRNA) expression, DNA methylation, can be explored to improve the accuracy of patient classification models [4]. Each of these data is termed ‘omic’ (genomics, transcriptomics, methylomics, respectively). The objective here is to identify groups with similar molecular characteristics.

Integrative clustering of several omics data for the same set of samples can disclose more precise structures that are not exposed by examining a single omic data. By exploiting the information present in multiple omics, clustering techniques can obtain better performance compared to a single omic. Some of the advantages of clustering based on multiple omics are given as follows: (i) multi-omics clustering reduces the effect of noise in the data, (ii) each omic can reveal structures that are not present in other omics, (iii) different omics can unfold different cellular aspects.

A major difficulty of cluster analysis is the selection of best clustering algorithm for a given data set [5].Many omic datasets possess heterogeneous structures whereas most of the existing clustering algorithms search for homogeneous structures from a dataset. The problem of algorithm selection for clustering datasets having heterogeneous structures can be addressed by combined use of cluster ensemble and multi-objective clustering techniques [6].

Recently, Li et al. [7] proposed a novel method of combining multi-objective optimization (MOO) with integrated decision making (IDM) to address the problem of combined heat and power economic emission dispatch. Authors used a two-stage approach. In the first stage, *θ*−dominance based evolutionary algorithm is used to generate Pareto-optimal front of the model. In the second stage, using fuzzy c-means clustering, the obtained Pareto-optimal solutions are clustered to identify the best compromise solutions using grey relation projection.

In this paper, the clustering problem is formulated as an optimization problem where different cluster quality measures are used as the objective functions. We have introduced a multi-objective based multi-view cluster ensemble algorithm (*enAMOSA*, in short), which simultaneously uses the concepts from both cluster ensemble and multi-objective based multi-view clustering algorithms. The key idea is to minimize problems associated with cluster analysis, as well as to overcome the limitations of multi-objective based multi-view clustering and cluster ensemble methods when they are used separately. Here ensemble is not used as a late-integration technique, but it is used as a perturbation operator for generating new solutions based on the selected parent solutions. Throughout this paper, omic is termed as view and multi-omic as multi-view in the context of algorithms. An overview of the proposed method is given below:

- *enAMOSA* conducts multi-view based multi-objective clustering by first identifying different partitions from the same data set using different views. To capture the goodness of an individual clustering generated using a single view, an internal cluster validity index, *conn*-XB index [8], is used. The values of the *conn*-XB index for different partitions obtained using varying views are simultaneously optimized along with agreement index [9]. Agreement index measures the agreement among multiple partitions obtained using different views in a new way. A special perturbation operator is used which replaces the traditional mutation operator. This operator uses an ensemble method along with the initial population for generating new diverse solutions. Finally, the partitions obtained on multiple views are combined to generate a single solution.
- A large number of experiments are conducted to illustrate the efficacy of different components of the proposed *enAMOSA* algorithm. We have developed several baseline methods by generating all possible combinations of the base partitions used in the experiment. These baselines are explained in detail in the later sections of the paper. To further demonstrate the effectiveness of the proposed perturbation operator, we have also compared results of *enAMOSA* with another version of multi-view AMOSA where normal perturbation operator is used (the perturbation operator used in [9]) and ensemble technique is used separately for combining the final Pareto optimal solutions generated by different clustering algorithms.
- The developed algorithm is tested on 13 genomic datasets. Results are compared with those obtained by baseline algorithms and existing state-of-the art models.

**Fig 1:**
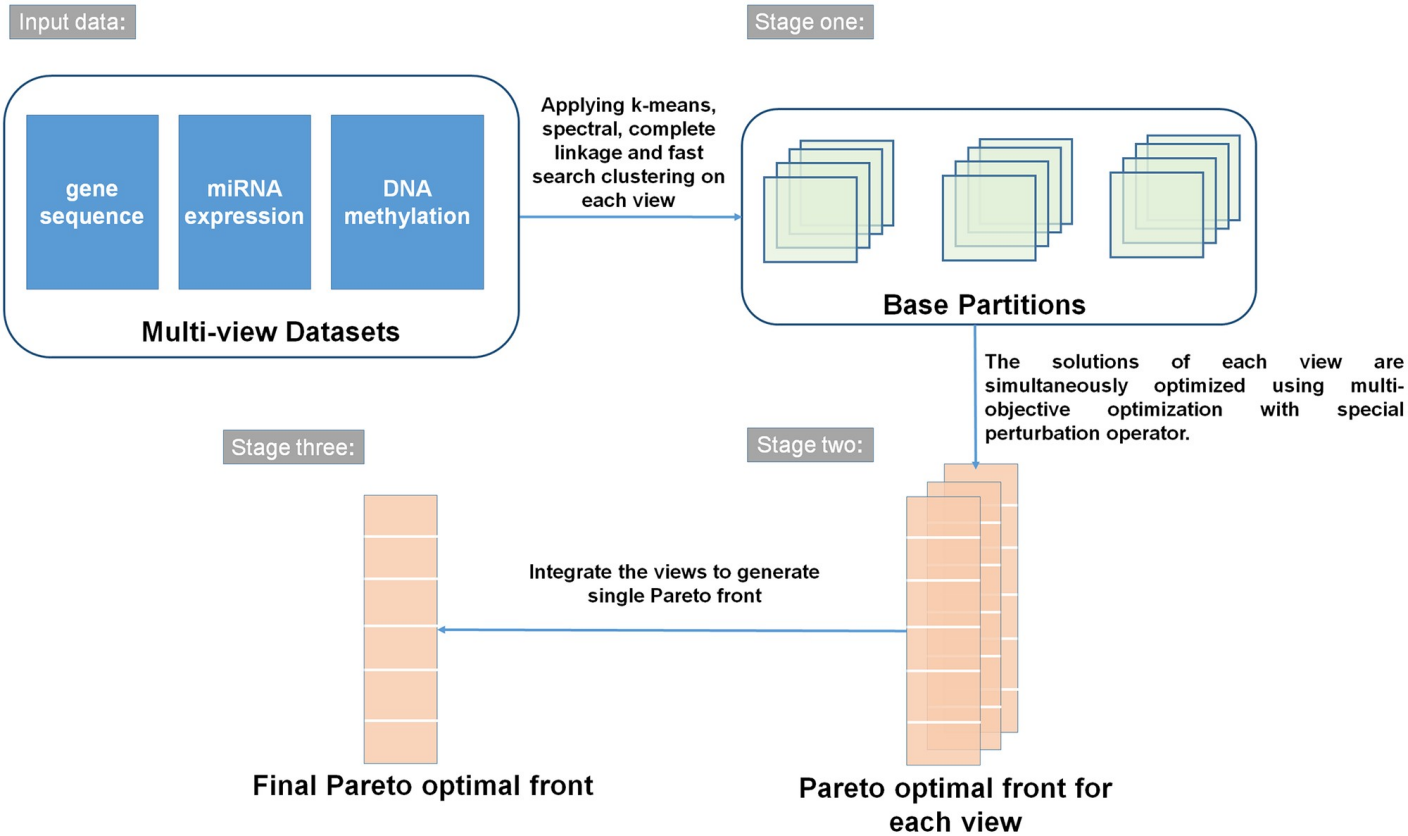
Overview of the proposed algorithm *enAMOSA*.

The overall steps of the proposed algorithm are shown in Fig 1.

Some of the contributions of our proposed methodology are as follows:

- To the best of our knowledge, this work is the first multi-objective based multi-view approach for capturing heterogeneous structures from multi-omics data in the field of patient classification.
- A new perturbation operator is designed by combining the concepts of both multi-objective multi-view clustering and cluster ensemble. It improves the robustness of the proposed algorithm to deal with data having different types of clusters.
- The algorithm is capable of capturing heterogeneous structures within a view and also amongst different views.
- In our proposed algorithm, different views can be clustered using different clustering algorithms. Further, the views can have a different number of clusters. To the best of our knowledge, previous multi-view multi-objective algorithms, like MvAMOSA [9], allow different views to be clustered by the same clustering algorithm and also restrict all the views to have the same number of clusters.

## Background

In the literature, several semi-supervised or supervised classification methods [10–12] are developed for cancer diagnosis. These classification techniques classify tumor samples in cancer dataset as malignant or benign or any other sub types [13]. But it is not always possible to obtain labeled tissue samples. For example, real life gene expression datasets in Ref. [14] or microRNA datasets in Ref. [15] are some unlabeled datasets. Hence, application of supervised classification techniques in cancer classification problem is difficult due to unavailability of labeled data. Thus clustering techniques become popular in solving different problems from bioinformatics domain. Multiple molecular profiling data can be collected for the same individual. Exploiting these data separately and then combining them can significantly improve the clinically relevant patient subclassifications [16].

This section discusses existing works on multi-view clustering, cluster ensemble techniques, drawbacks of the state-of-the art models and motivation of the work.

### Existing works on multi-omics/view clustering methods

The increase of multi-modal datasets in real-world applications has raised the interest in multi-view learning [17].

Based on the algorithmic approach multi-view clustering methods can be broadly classified into three categories; (i) Early integration, (ii) Late integration, and (iii) Intermediate integration.

Early integration approach is the simplest amongst all. In this approach, at first, all the different views are concatenated to form a single large dataset with features from multiple views. The resulted dataset is clustered using any single-view clustering method. However, this approach has some major drawbacks. Firstly, it causes a significant increase in the data dimension which is a challenge for clustering algorithms. Secondly, it ignores different distributions present in different views of the dataset. LRACluster [18] and Structured sparsity [19] are some of the methods which use early integration approach. LRACluster [18], uses a latent representation of the samples to determine the distribution of numeric, count and binary features. It optimizes a convex objective and provides a globally optimal solution. Structured sparsity [19] method concatenates the views and applies a weighted linear transformation for clustering. The features that do not contribute to the cluster structure are assigned with low weights.

In late integration approach, each view of the dataset is clustered separately using a single-view algorithm. Here, each view can be clustered using different clustering algorithms. Finally, the clusters from different views are integrated to form combined global clusters. COCA [20] and PINS [21] are examples of methods using this approach. PINS [21] uses a connectivity matrix to integrate clusters of different views. This algorithm first adds some Gaussian noise to the data, the cluster number is chosen in a way that clustering is robust to the perturbation. Serra et al. [16], proposed a multi-view approach, MVDA, for identifying different clinically relevant patient-subclasses by combining the information present in multiple high-throughput molecular profiling data sets generated by omics technologies.

Intermediate integration approach involves the following; (i) methods where views are integrated using similarity/distance, (ii) methods that use joint dimension reduction for different views and (iii) methods using statistical modelling of the views.

Chikhi [22], proposed a generalized spectral clustering algorithm, Multi-View Normalized Cuts (MVNC). It is a two-step approach. Initially, the spectral clustering is applied on the dataset followed by a local search to refine the initial clustering. Similarity Network Fusion (SNF) [23] is another similarity-based method which constructs a similarity network for each view separately. Using an iterative process these networks are fused together. Regularized Multiple Kernel Learning with Locality Preserving Projections (rMKL-LPP) [24], performs dimensionality reduction on different views such that similarities amongst the samples are preserved in low dimensions. Subsequently, K-means is applied to this low dimensional representation. Zhang et al. in [25], proposed CMVNMF (Constrained Multi-View clustering based on NMF). It is an extension of the NMF model where different views can contain different samples, but certain samples from different views are constrained to be in the same cluster. iCluster [26] utilized a joint latent-variable model to detect the grouping structure from multi-omics data. iCluster+ [27], an extension of iCluster, includes different models but maintains the idea of iCluster that data originates from a low dimension. The latest extension is iClusterBayes [28]. This method uses Bayesian regularization and is much faster compared to its previous variants. In [29], authors proposed an parameter-free clustering models, Adaptively Weighted Procrustes technique, for multiview clustering. Authors in [30], proposed a self weighted multiview clustering technique (SwMC).

### Existing works on cluster ensemble

Cluster ensemble is a technique of deriving a better clustering solution from a set of candidate clustering solutions [5, 31]. A cluster ensemble algorithm can be presented as a two step approach: (i) a diverse set of base partitions are generated; and (ii) these partitions are combined to form a single consensus partition. Depending on the type of base partitions, cluster ensemble is of two types, viz., homogeneous and heterogeneous. When base partitions are obtained from same clustering algorithm, it is called homogeneous and in contrast if base partitions are obtained from different clustering algorithms, it is called heterogeneous. Based on the type of consensus function used, the existing approaches of cluster ensemble are mainly categorized under co-association, graph/hyper-graph partitioning, mutual information or re-labeling [6].

In [32], authors formalized the cluster ensemble problem as a combinatorial optimization problem in terms of shared mutual information. They have proposed three algorithms: MCLA (meta- clustering algorithm), cluster-based similarity partitioning algorithm (CSPA) and hyper-graph partitioning algorithm (HGPA). Depending on the mutual information shared, a consensus function can be applied to select the best partition amongst those produced by these three algorithms.

Based on the base partitions, the CSPA algorithm constructs a similarity matrix. Values in the matrix denote the fraction of partitions where two objects belong to the same cluster. Further, a similarity-based clustering algorithm is applied on this matrix to generate the consensus partitioning.

In HGPA algorithm, a hypergraph is constructed by representing base partition clusters as hyper-edges of the graph. This hypergraph is partitioned by cutting with a minimal number of hyper-edges.

In MCLA algorithm, a meta-graph is constructed, where each base partition cluster forms the vertex. Similarity between the vertices represents the edge weights of this graph. Vertices belonging to the same partition do not have edges. On partitioning the meta-graph, the clusters belonging to the same group are considered correspondents. The objects are assigned to the meta-clusters they are strongly associated with, generating the consensus partition.

In the HBGF (Hybrid bipartite graph formulation) HBGF [33], a bipartite graph is constructed from the set of base partitions. Objects and clusters are simultaneously modeled as vertices of the graph. In the end, a graph partitioning algorithm is applied on the generated bipartite graph. The resulting division of the objects is the consensus partitioning.

### Drawbacks of the existing literature

In the field of patient sub-classification, multi-view data from multiple omics technologies can be obtained for same individual. The clinically relevant patients sub-classification can be significantly improved by combining these data, rather than exploiting them separately. However, by and large, multi-view clustering approaches have not penetrated bioinformatics yet [34]. The existing multi-view based classification techniques for patient sub-classification suffer from the following drawbacks:

1. Existing multi-view clustering problems are mostly solved as single objective optimization problems. A single quality measure for partitioning is optimized implicitly or explicitly using various paradigms of unsupervised single-view learning. Initially different views of the dataset are partitioned and later the agreement between the partitions obtained on different views is optimized. But instead of treating these two objectives (goodness of partitions obtained using individual views and agreement among-st partitions) separately, it is better to optimize them simultaneously for capturing better partitioning structures among the views.
2. The existing multi-view based approaches applied for patient sub-classification problem are very simple in structure and cannot effectively identify more than one relevant structures of the datasets.
3. Multi-objective clustering algorithms can identify different alternative partitionings of a dataset after a single execution. But as the number of alternatives increases, the analysis becomes harder.
4. In the patient-stratification problem, cluster ensemble is mostly used during view integration. But, the literature lacks the use of any multi-view multi-objective algorithm combinedly with an ensemble technique rather than separately, to capture fine-structures present among different views.
5. Most of the existing multi-view algorithms are designed to capture homogeneous structures among multiple views.
6. Existing multi-view multi-objective algorithms allow the same clustering algorithm for partitioning the data over multiple views of the sample and also restrict the views to have the same number of clusters.

### Motivation

The general aim of any multi-view clustering is to improve the cluster quality in each view and to increase the agreement between multiple partitionings obtained using individual views. By nature it is a multi-objective optimization problem with two types of objectives, cluster quality over different views and agreement between multiple views, to be optimized simultaneously. Further, multi-omics datasets exhibit complex structures, difficult for single-objective based clustering algorithms to capture. Although the multi-objective approach offers a set of alternative structures of the dataset, as the number of alternatives increases, the analysis becomes harder. All these motivated us to develop a new multi-objective based multi-view algorithm with a unique ensemble based perturbation operator that is capable of capturing the fine-tuned structures in multi-omic datasets.

## Problem formulation

The multi-view cluster ensemble problem is formulated as a multiobjective optimization problem.

- Given:
  - A multi-view dataset containing *V* views and *n* number of samples $S=\{{\bar{x}}_{1},{\bar{x}}_{2},\ldots ,{\bar{x}}_{n}\}$,
  - *d*_*m*_ is the number of features in the *m*^*th*^ view, and *D*^*m*^ is the *n* × *d*_*m*_ matrix representing the *m*^*th*^ view.
  -
$D_{ij}^{m}$ is the *j*^*th*^ feature of the *i*^*th*^ sample in the *m*^*th*^ view.
  - Concatenation of the *V* views produces matrix *D* of size *n* × *d*, where $d=\sum_{m=1}^{V}d_{m}$ is the total number of features.
  - A set of base clustering algorithms, *CA*_1_, *CA*_2_, …, *CA*_*p*_.
  - A set of objective functions
$$CV_{1},CV_{2},\ldots ,CV_{m},AI,$$
    where each *CV*_*i*_ is a cluster validity index measured on the partitioning obtained after considering only view *m* for the given data set, and *AI* is used for measuring the agreement between the partitions obtained for different views.
- Find:
  - A consensus partitioning (U) generated by ensembling the outputs of clustering algorithms, *CA*_1_, *CA*_2_, …, *CA*_*p*_, satisfying all views
    - The set of samples, *S*, is divided into *K* clusters, {*U*_1_, *U*_2_, …, *U*_*K*_}
    -
$U_{i}=\left\{{\bar{x}}_{1}^{i},{\bar{x}}_{2}^{i},\ldots ,{\bar{x}}_{n_{i}}^{i}\right\}$ ; *n*_*i*_: number of samples in cluster *i*; ${\bar{x}}_{j}^{i}$: *j*th sample of cluster *i*.
    -
$\cup_{i=1}^{K}U_{i}=S$ and *U*_*i*_ ∩ *U*_*j*_ = ∅ for all *i* ≠ *j*.
  - which simultaneously optimizes the objective functions. The simultaneous optimization of these objectives produces a Pareto optimal front.

## Materials and methods

### *enAMOSA*: Ensemble based multi-view archived multi-objective simulated annealing

This section discusses about the proposed multiobjective based multi-view cluster ensemble approach, namely *enAMOSA*.

To overcome the difficulties of traditional clustering algorithms, *enAMOSA* combines characteristics of cluster ensemble and multi-view based multi objective clustering methods. *enAMOSA* comprises of three main steps: (1) generation of diverse set of base partitions for each view, (2) determination of an ensembled partitioning considering the multiple base partitions and (3) finally generating a consensus partitioning satisfying different views. The proposed algorithm differs from traditional ensemble approach in two ways. Firstly, instead of producing a single consensus partitioning, it produces a set of consensus partitionings. In fact, the set of solutions can contain partitionings that are combinations of other partitionings, or partitionings of high quality that already appeared in the set of individual partitionings. Secondly, it is an iterative process. For each iteration, it combines pairs of partitionings for each view and then the views are integrated to generate a new solution for evaluation. The steps involved in *enAMOSA* are shown in Algorithm 1.

The calculation of dominance among the solutions is the same as in AMOSA [35]. In the Algorithm 1, temperature (*temp*) plays a significant role in calculating the probability of acceptance of a solution.

**Algorithm 1:** Algorithm for enAMOSA

 **Initialize:** *iter*, *SL*, *HL*, *T*_*min*_, *T*_*max*_, *no*_*views*, *α*, *temp* = *T*_*max*_

**1 begin**

**2**  Initialize *pool* with solutions from k-means, complete linkage, fast search clustering and spectral clustering.

**3**  **for** *i = 1 to pool_size* **do**

**4**   **for** *j = 1 to no_views* **do**

**5**    ComputeFitnessconnXB(*pool*[*i*], *j* /* Compute conn-XB for each view */

**6**   **end**

**7**   ComputeFitnessAI(*pool*[i])

**8**  **end**

**9**  Compute dominance of the solutions in *pool*.

**10**  Initialize *Archive* with the non-dominated solutions of *pool*

**11**  *current* = random(*Archive*)

**12**  **while** *temp* ≥ *T*_*min*_ **do**

**13**   **for** *gen* = 1 to *iter* **do**

**14**    *new*_*pt* = perturb *current*

**15**    **for** *j* = 1 to *no*_*views* **do**

**16**     ComputeFitnessAI(*new*_*pt*[*j*], *j*)

**17**    **end**

**18**    ComputeFitnessAI(*new*_*pt*)

**19**    Compute dominance of *current* and *new*_*pt*

**20**    Update *Archive* and *current*

**21**   **end**

**22**   *temp* = *α* × *temp*

**23**  **end**

**24**  Pareto_front = CombineViews(*Archive*)

**25 end**

#### Generation of base partitions

To generate the initial solutions (called base partitions), four different clustering techniques (called base clustering algorithms), hierarchical (complete linkage) [36], K-means, fast search [37] and Spectral clustering, are applied on each view of the given dataset. These four algorithms used belong to different categories of the clustering algorithm, like, K-means represents the centroid models, hierarchical represents the connectivity model and, spectral and fast search represent the density based model of clustering. The more diverse the base algorithms, higher the chances of capturing differently shaped clusters of the data set. It is essential to have different types of partitionings in the initial archive so that *enAMOSA* can receive as much information as possible to find an optimal number of possible existing structures.

The choice of clustering algorithms for generating base partitions are not merely restricted to these four clustering algorithms only, but other clustering algorithms can also be used.

The number of clusters (K) which will be given as an input to the base clustering algorithm (∧) is determined randomly. The number of clusters is varied over the range *K*_*min*_ to *K*_*max*_. Here, the value of *K*_*min*_ = 2 and $K_{max}=\sqrt{n}$, where *n* denotes the number of samples. A value K is selected randomly between the range *K*_*min*_ and *K*_*max*_ with uniform probability. ∧ is applied to the data set with the number of clusters = K varying the views.

But fast search [37] is a density-based clustering and parameters are determined automatically from the corresponding views. This algorithm does not consider the number of clusters as input. It automatically determines the number of clusters from any given dataset.

At the end of this step, we have a set of base partitions for each view.

#### Archive initialization

For each view, we compute the dominance of the base partitions obtained in previous step. A set of non-dominated solutions are generated from each view. The archive is initialized with these non-dominated solutions.

The initial population of the archive in *enAMOSA* is not generated randomly, as is done for most of the AMOSA based clustering techniques. Instead, it is composed of a set of base partitions, *π*_1_, generated by running a diverse set of conceptually different algorithms.

#### String representation

In order to represent the initial partitioning solutions generated by different clustering algorithms, membership matrix based representation scheme is used.

For example, if K-means is executed on V different views with the corresponding set of attributes with the number of clusters = K, then for each case, a membership matrix, *Mem* of size *K* × *n* is obtained as follows:

$$\begin{array}{c} Mem_{ij}=\{\begin{array}{cc} 1 & \;\text{if}\;{\bar{x}}_{j}\in U_{i}\\ 0 & \;\text{otherwise} \end{array} \end{array}$$

Here, ${\bar{x}}_{j}$ denotes the *j*^*th*^ data point and *U*_*i*_ denotes cluster *i*. *Mem*_*ij*_ denotes the membership value of the *j*^*th*^ data point for the *i*^*th*^ cluster.

**Fig 2:**
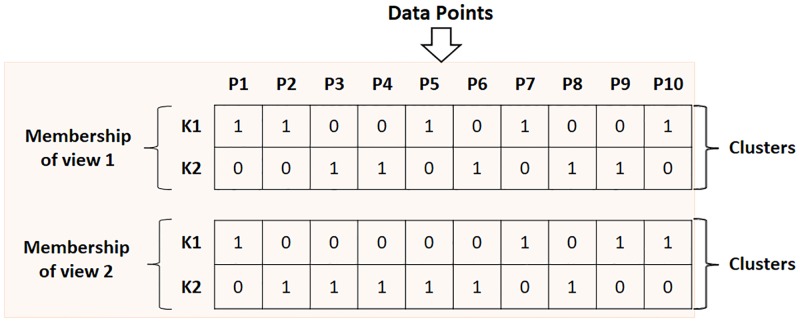
Membership matrices represented in a solution. In the example, there are two views, two clusters, and a data set of ten points.

Suppose the data set is having total V views and the clustering algorithm ∧ is selected to be executed on the data set. Then for a given view, a membership matrix of size *K* × *n* is generated. Total *V* such membership matrices of size *K* × *n* are encoded in the string. Thus length of the string is *V* × *K* × *n*. Fig 2 shows an example of the proposed string representation. All the strings of the archive are initialized in the above way.

#### Perturbation operator

The special perturbation operator uses ensemble method along with initial population for generating new solutions.

**Fig 3:**
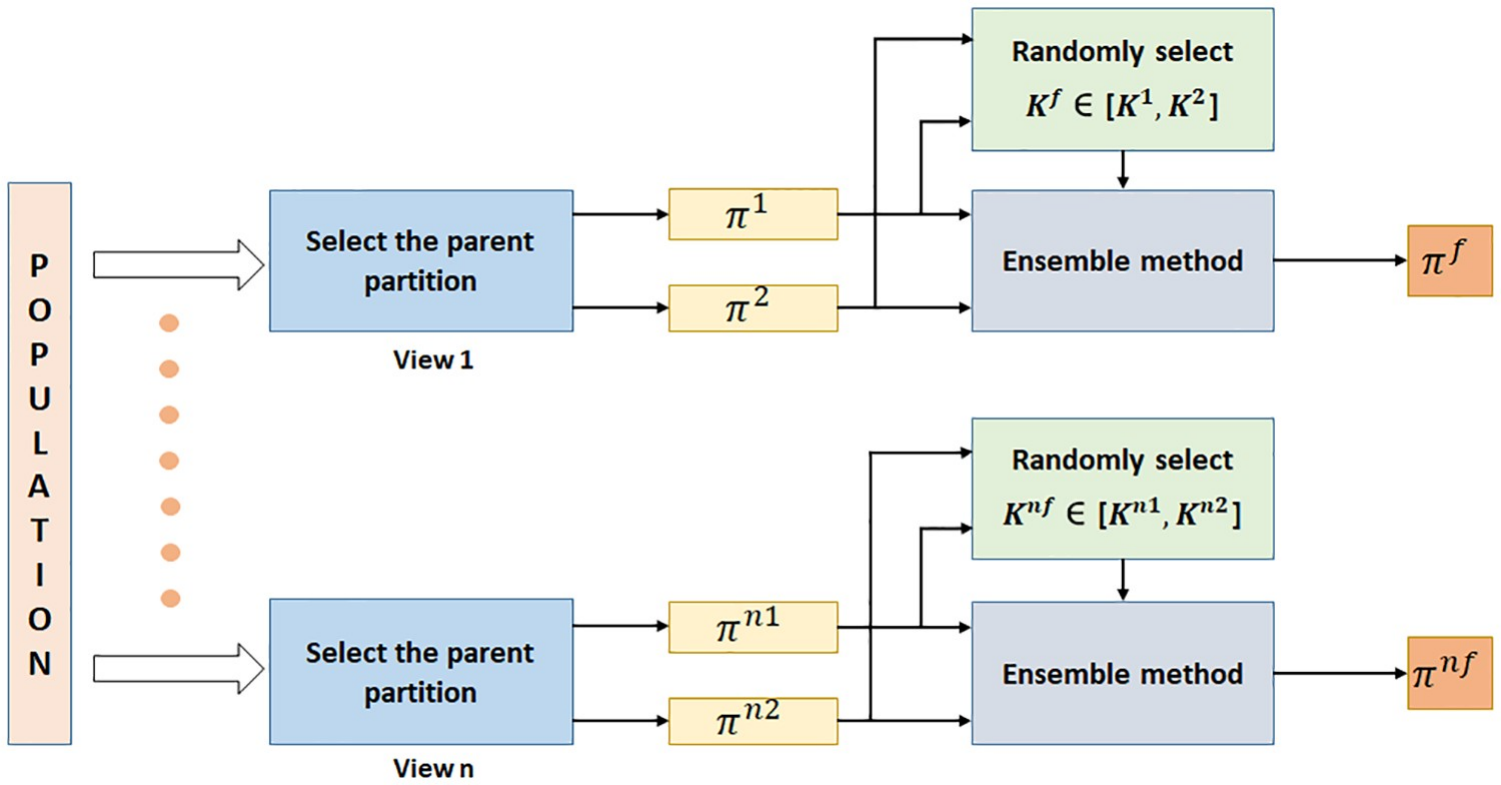
*enAMOSA* perturbation operator.

This operator finds the consensus partitioning between a pair of selected parents, for each individual view. Any existing cluster ensemble method can be used in *enAMOSA* as the perturbation operator. The idea is to generate new good-quality solutions which are combinations of previous two solutions. First, two parents are randomly selected from the archive to be combined. The combination is done for individual views. Ensemble based operator is applied on the membership matrices present in two selected solutions for a given view. Let $\pi_{1}^{1}$ and $\pi_{1}^{2}$, be the membership matrices of two selected solutions, respectively, with $K_{1}^{1}$ and $K_{1}^{2}$ number of clusters ($\pi_{1}^{2}$ means second selected parent from view 1 and similarly for others). Let the ensembled solutions be represented by $\pi_{1}^{F}$ and $\pi_{2}^{F}$. The number of clusters $K_{1}^{F}$ for partition $\pi_{1}^{F}$ is chosen randomly in the interval $\left[K_{1}^{1},K_{1}^{2}\right]$. Second, the parents are combined using ensemble method. The consensus partition generated has $K_{1}^{F}$ clusters. Illustration of the operator is given in Fig 3. The operator is briefly described in Algorithm 2.

Here, CSPA (cluster-based similarity partitioning algorithm) technique [32] is used as underlying ensemble method. The operator worked as follows:

- Let the selected clustering solutions be: *π*^1^ and *π*^2^ with *K*^1^ and *K*^2^ number of clusters, respectively. Let the corresponding partitionings be $U_{1}^{1},U_{2}^{1},U_{3}^{1},\ldots ,U_{K^{1}}^{1}$ and $U_{1}^{2},U_{2}^{2},U_{3}^{2},\ldots ,U_{K^{2}}^{1}$ corresponding to the solutions, *π*^1^ and *π*^2^, respectively.
- For each partitioning solution, an adjoint matrix, $A_{n\times n}^{k}$, *k* = 1, 2 is generated as follows:
  $$\begin{array}{c} A_{ij}^{k}=\{\begin{array}{cc} 1 & \;\text{if}\;\text{points}\;i\;\text{and}\;j\;\text{belong}\;\text{to}\;\text{the}\;\text{same}\;\text{cluster}\\ 0 & \;\text{otherwise}\; \end{array} \end{array}$$ (1)
- A new similarity matrix, *Sim*_*n* × *n*_ is computed as follows:
  $$\begin{array}{c} Sim_{ij}=sum_{k=1}^{2}A_{ij}^{k} \end{array}$$ (2)
  This similarity matrix is used for clustering the data set using any standard similarity-based clustering algorithm like hierarchical clustering technique. In general an induced similarity graph (vertex = object, edge weight = similarity) approach using METIS [38] can be used along with the newly generated similarity matrix.

The above ensemble based operator is applied for individual views separately.

**Algorithm 2:** Algorithm for new perturbation operator.

 **procedure:** perturb(Element)

**1 begin**

**2**  *Element*_2_ = random(*Archive*) /*Select a random solution from *Archive* except Element*/

**3** **for** *i* = 1 to *no*_*views* **do**

**4**  *K*_1_ = no_of_clusters(*Element*[*i*])

**5**  *K*_2_ = no_of_clusters(*Element*_2_[*i*])

**6**   *K*_*F*_ = randi(*K*_1_, *K*_2_) /*Generate integers in range *K*_1_ and *K*_2_*/

**7**   *new*_*pt*[*i*] = CSPA(*Element*[*i*], *Element*_2_[*i*], *k*_*F*_)

**8**  **end**

**9**  return *new*_*pt*

**10 end**

#### Objective functions

The optimization framework uses two objective functions: (i) Agreement Index [9] for measuring the agreement between partitions obtained from different views, and (ii) Connectivity based XB-Index or *conn*-XB Index [8].

#### Agreement index

Agreement Index [9] is used for measuring the agreement between partitions obtained from different views.

$$\begin{array}{c} AI_{v1,v2}=\frac{n_{a}+1}{n_{d}+1},n_{a}=\sum_{i=1}^{n}\sum_{j=1}^{n}I_{A_{ij}^{v1},A_{ij}^{v2}}\\ n_{d}=n^{2}-n_{a}\\ I_{A_{ij}^{v1},A_{ij}^{v2}}=\{\begin{array}{cc} 1 & \;\text{if}\;A_{ij}^{v1}=A_{ij}^{v2}\\ 0 & \;\text{otherwise}\; \end{array} \end{array}$$

Here *A*^*v*1^ and *A*^*v*2^ are adjoint matrices of the views *v*1 and *v*2 respectively. The final *Agreement index* for the total partitioning is calculated as follows:

$$\begin{array}{c} AI_{total}=\frac{\sum_{l=1}^{V}\sum_{m=1,l\neq m}^{V}2\times AI_{v_{l},v_{m}}}{V\times (V-1)} \end{array}$$

Here, *n* total samples in the dataset and *V* is the number of views.

#### *connected*-XB or *conn*-XB Index

In [8], authors have developed connectivity based XB-Index. The definition of this index follows the formulation of popular XB-Index [39].

$$\begin{array}{c} conn-XB=\frac{\sum_{i=1}^{K}(\sum_{\bar{x}\in U_{i}}d_{short}\left(\bar{x},{\bar{z}}_{i}\right))}{n(min_{i,k=1\ldots K,i\neq k}d_{short}\left(\bar{z_{k}},{\bar{z}}_{i}\right))} \end{array}$$ (3)

$d_{short}\left(\bar{x},{\bar{z}}_{i}\right)$ is the shortest distance between two points, $\bar{x}$ and ${\bar{z}}_{i}$, along the relative neighborhood graph [8]. It measures the connectivity between two points. If two points are connected / a path exists between these two points along the relative neighborhood graph (RNG) then *d*_*short*_ value will be low. Here *U*_*i*_ denotes the cluster *i*, ${\bar{z}}_{i}$ is the medoid of cluster *i*, *n* is the size of the whole data set and ${\bar{z}}_{j}$ denotes the cluster *j*. The objective is to lower the value of *conn-XB* index in order to obtain good partitioning.

A solution encodes total *V* number of membership matrices/partitionings. For each such membership matrix/partitioning, the value of *conn-XB* is calculated to measure the goodness of this partitioning. Let the values be *conn*−*XB*_1_, *conn*−*XB*_2_, …, *conn*−*XB*_*V*_. Then the objective functions corresponding to a single solution are

$$\left\{conn-XB_{1},conn-XB_{2},\ldots ,conn-XB_{V},\frac{1}{AI}\right\}$$

*enAMOSA* simultaneously optimizes these (*V* + 1) number of objective functions.

**Algorithm 3:** Algorithm for combining views

**procedure:** CombineViews(*Archive*)

**1 begin**

**2**  **for** *i* = 1 *to archive size* **do**

**3**   Initialize each element of *temp*_*sum* with 0 /*Size of *temp*_*sum* is *sample* × *sample* */

**4**   **for** *j* = 1 to *no*_*views* **do**

**5**    *adj* = GenerateAdjMatrix(*Archive[i][j]*) /*Generate adjacency matrix for each view*/

**6**    *temp*_*sum* = *temp*_*sum* + *adj*

**7**   **end**

**8**   Initialize each element of *new*_*adj* with 0 /*Size of *new*_*adj* is *sample* × *sample* */

**9**   **for** *k* = 1 to *sample* **do**

**10**    **for** *l* = 1 to *sample* **do**

**11**     **if** $temp\_sum\left[k\right]\left[l\right]>\frac{no\_views}{2}$ **then**

**12**      *new*_*adj*[*k*][*l*] = 1

**13**     **end**

**14**    **end**

**15**   **end**

**16**    Generate clusters from *new*_*adj* matrix.

**17**  **end**

**18 end**

#### Consensus function for view combination

At the end of the execution of *enAMOSA*, we get a set of non-dominated solutions on the final archive. The psedu code is given in Algoriyhm 1. Each of these solutions encodes total *V* number of membership matrices. A new late integration method is proposed to combine the membership matrices present in a single solution. A consensus partitioning is obtained satisfying all the available views. So, in order to get a consensus partitioning, initially the common points of different clusters present in different partitionings obtained using different views are identified. This is achieved by majority voting scheme. If a pair of points cluster together in majority of the views then in the final partitioning they will also be grouped together. Likewise all the pairs of data points are evaluated. If some points are not assigned to any group (this situation may occur if even number of views are used and a tie occurs) then in the final partitioning, these points are assigned to the group of their nearest neighbors. The process is illustrated below:

- Let the adjoint marices of the partitionings present in a string corresponding to different views be denoted by *A*^*k*^ where *k* = 1…*V*, *V* = *totalnumberofviews*. Then a new adjoint matrix, *A*^*sum*^ is computed as follows:
  $$\begin{array}{c} A_{ij}^{sum}=\sum_{k=1}^{V}A_{ij} \end{array}$$ (4)
- Now a new matrix *A*^*new*^ is generated as follows:
  $$\begin{array}{c} A_{ij}^{new}=\{\begin{array}{cc} 1 & \;\text{if}\;A_{ij}^{sum}>\lceil \frac{V}{2}\rceil \\ 0 & \;\text{Otherwise}\; \end{array} \end{array}$$
- The matrix *A*^*new*^ is used to generate the final partitioning. Following a link based approach, connected components of the matrix *A*^*new*^ are identified. Points are considered as vertices and the points, (*i*, *j*), whose $A_{ij}^{new}=1$ are connected by an edge. The connected components of this graph are treated as initial clusters. Let total number of clusters be *K*.
- For rest of the points which are not part of any of the clusters extracted in the previous step, cluster assignment is done as follows. Any point ${\bar{x}}_{i}$ will be assigned to *k*th cluster where:
  $$\begin{array}{c} k=\overset{K}{\underset{k=1}{\mathrm{argmin}}}\min_{j=1}^{n_{k}}d_{short}\left({\bar{x}}_{i},{\bar{x}}_{j}^{k}\right) \end{array}$$ (5)
  Here *K* denotes total number of clusters/connected components identified from the previous step. *n*_*k*_ denotes the number of points in the *k*th connected component/cluster, ${\bar{x}}_{j}^{k}$ denotes *j*th point of the *k*th cluster and $d_{short}\left({\bar{x}}_{i},{\bar{x}}_{j}^{k}\right)$ denotes the shortest distance [8] between ${\bar{x}}_{i}$ and ${\bar{x}}_{j}^{k}$.
- Finally a partitioning will be obtained where all the points are part of some clusters. This partitioning is reported as the final consensus partitioning for that particular solution.
- For each solution present in the archive, a single consensus partitioning is obtained. If *archive*−*size* = *N*, then *N* such consensus partitionings will be generated.

### Theoretical analysis

#### Complexity analysis

In this section, the time complexity of *enAMOSA* is discussed. The basic steps and their complexities are as follows:

1. Initialization of Archive: *O*(*SL*)
2. Domination status between two solutions: *O*(*M*), where *M* = number of objectives
3. Domination status between a single solution and archive elements: *O*(*M* × *SL*)
4. Complexity of perturbation (here we used CSPA): *O*(*n*^2^ × *r* × TotalIter), where *n* = no. of samples, *r* = no. of clusters
5. Single linkage clustering: *O*(*SL*^2^ × log(*SL*))
6. Clustering is done:
  - during initialization if *NDom* > *SL*, where *NDom* = number of non-dominated solutions.
  - after each |*SL*−*HL*| number of iterations
  - if *Archive*−*size* > *HL* in the end
  Clustering is executed for $\frac{TotalIter}{\left(SL-HL\right)}+2$ times.
  $$\begin{array}{c} \text{Clustering}\;\text{complexity}\;=\;O(\frac{TotalIter}{\left(SL-HL\right)}\times SL^{2}\times \log\left(SL\right)) \end{array}$$

Final time complexity of *enAMOSA* is

$$\begin{array}{c} TotalIter\times (SL+M+M\times SL+n^{2}\times r+\frac{1}{\left(SL-HL\right)}\times SL^{2}\times \log\left(SL\right)) \end{array}$$

Let *HL* = *N*, where *N* = size of Archive and *SL* = *γ* × *HL*, *γ* ≥ 2

$$\begin{array}{c} TotalIter\times (\gamma \times N+M+M\times \gamma \times N+n^{2}\times r+\frac{\gamma^{2}}{\left(\gamma -1\right)}\times N\times \log\left(\gamma \times N\right)) \end{array}$$

The final time complexity of *enAMOSA* is:

$$\begin{array}{c} O(TotalIter\times \left(n^{2}\times r+N\times \left(M+\log\left(N\right)\right)\right)) \end{array}$$ (6)

#### Convergence analysis

In the proposed algorithm *enAMOSA*, we have simultaneously optimized two objectives, *conn*-XB Index [8] and Agreement index [9].

*conn*-XB Index [8] follows the formulation of popular XB-Index [39]. It measures the ratio between the cluster compactness and cluster separation. Xie-Beni validation index behaves convex when the samples are around the optimal values for the centroids [40]. Similarly, *conn*-XB Index [8] behaves convex under same condition.

Agreement index [9] measures the agreement between partitions obtained using different views. It is given by the following equation:

$$\begin{array}{c} AI=\frac{n_{a}+1}{n_{d}+1} \end{array}$$ (7)

where, *n*_*a*_ = number of pairs of samples occurring together in both the views.

*n*_*d*_ = number of pairs of samples not occurring together in different views.

If there are n number of samples then,

$$\begin{array}{c} n_{d}=n^{2}-n_{a} \end{array}$$ (8)

Now, replacing Eq 8 in Eq 7, we have

$$\begin{array}{c} AI=\frac{n_{a}+1}{n^{2}+1-n_{a}} \end{array}$$ (9)

The value of *n*_*a*_ is 0 ≤ *n*_*a*_ ≤ *n*^2^, 0 when all pairs disagree and *n*^2^ when all pairs agree.

$$\begin{array}{c} \forall x\in n_{a},\frac{1}{n^{2}+1}\leq AI\left(x\right)\leq n^{2}+1 \end{array}$$ (10)

Hence, *AI* is a monotonically increasing function in the range of *n*_*a*_.

*enAMOSA* follows the formulation of AMOSA [35]. The acceptance probability is crucial for the behavior of the simulated annealing. *enAMOSA* adopts a dynamic acceptance which is dependent on the domination status [35]. It is given by:

$$\begin{array}{c} P_{acc}=\frac{1}{1+\exp^{\frac{-\Delta E_{q,s,t}}{T}}} \end{array}$$ (11)

where, Δ*E*_*q*,*s*, *t*_ represents the change in energy state of state *q* and state *s* at given temperature *T*. The convergence proof of simulated annealing based multi-objective optimization is elaborately explained in [41].

All the above mentioned factors ensure the convergence of the proposed algorithm.

### Dataset collection and preparation

To evaluate the performance of the proposed algorithm we have used a total of 13 benchmark omic datasets. The details of the datasets are given in Table 1. The datasets are downloaded from the following repositories: The Cancer Genome Atlas (TCGA) https://tcga-data.nci.nih.gov/tcga/, NCBI GEO http://www.ncbi.nlm.nih.gov/geo and Memoral Sloan-Kettering Cancer Center (MSKCC)http://cbio.mskcc.org/cancergenomics/prostate/data/.

**Table 1:** Descriptions of datasets.

Dataset	Views	Total Features	Selected Features	Samples
*TCGA.BRC*	RNASeq	20510	4300	621
	miRNASeq	1046	220	
	DNA Methylation	4885	1125	
*OXF.BRC.1*	Gene Expression	21439	4500	349
	miRNA Expression	734	164	
	DNA Methylation	4885	1125	
*OXF.BRC.2*	Gene Expression	21439	4500	349
	miRNA Expression	734	164	
	DNA Methylation	4885	1125	
*MSKCC.PRA*	Gene Expression	26446	5300	151
	miRNA Expression	368	82	
	DNA Methylation	3894	858	
*TCGA.GBM*	Gene Expression	12042	2500	274
	miRNA Expression	534	110	
	DNA Methylation	5000	1200	
*TCGA.OVG*	Gene Expression	12043	2500	398
	miRNA Expression	800	190	
	DNA Methylation	5000	1200	
*TCGA.COAD*	Gene Expression	20351	4883	220
	miRNA Expression	705	170	
	DNA Methylation	5000	1200	
*TCGA.LIHC*	Gene Expression	20531	4792	367
	miRNA Expression	705	170	
	DNA Methylation	5000	1200	
*TCGA.LUSC*	Gene Expression	20531	4880	341
	miRNA Expression	705	170	
	DNA Methylation	5000	1200	
*TCGA.SKCM*	Gene Expression	20531	4884	448
	miRNA Expression	705	170	
	DNA Methylation	5000	1200	
*TCGA.SARC*	Gene Expression	20531	4617	257
	miRNA Expression	1046	241	
	DNA Methylation	5000	1150	
*TCGA.KIRC*	Gene Expression	20531	4880	183
	miRNA Expression	705	170	
	DNA Methylation	5000	1200	
*TCGA.AML*	Gene Expression	20531	4520	170
	miRNA Expression	705	168	
	DNA Methylation	5000	1198	

#### TCGA.BRC

This Breast cancer the dataset contains samples from patients with invasive tumors. It contains data for three views: miRNASeq(Level 3), RNAseq and DNA Methylation. Using PAM50 classifier [42, 43] patients were classified into four categories: Her2, Basal,LumA, LumB.

#### OXF.BRC.1

This Breast cancer [1][44] dataset contains data for three views: microRNA expression (GSE22220 accession number), mRNA (GSE22219 accession number) and DNA Methylation. Using PAM50 classifier [42, 43] patients were classified into four categories: Her2, Basal,LumA, LumB.

#### OXF.BRC.2

This Breast cancer [44] dataset contains data for three views: microRNA expression (GSE22220 accession number), mRNA (GSE22219 accession number) and DNA Methylation. Using clinical data also retrieved from the same source, patients were classified into four categories: Level1, Level2, Level3, Level4.

#### MSKCC.PRCA

This dataset contains samples from patients with prostate cancer tumors. It has three views: gene expression, miRNA expression and DNA Methylation. According to a study performed on this dataset [45], patients are grouped into two categories: first class is Tumor stage I and the second class is Tumor stage II, III and IV.

#### TCGA.GBM

Glioblastoma cancer the dataset has three views: gene expression, miRNA expression and DNA Methylation. As described in [46], patients are grouped into four categories: Classical, Mesenchymal, Neural and Proneural.

#### TCGA.OVG

Ovarian cancer dataset contain samples from patients with ovarian serous cystadenocarcinoma tumors. It has three views: gene expression, miRNA expression and DNA Methylation. Based on clinical stages, patients are grouped into four categories: class one: stage IA, IB, IC, IIA, IIB and IIC; class two: IIIA, IIIB and IIIC; class three: stage IV.

#### TCGA.COAD

Colon cancer dataset contain samples from patients suffering from Colon Adenocarcinoma (COAD). It has three views: gene expression, miRNA expression and DNA Methylation. Based on clinical stages, patients are grouped into four categories: class one: stage I, IA, IB, IC; class two: II, IIA, IIB and IIC; class three: III, IIIA, IIIB and IIIC; class four: stage IVA, IVB, IV.

#### TCGA.LIHC

Liver cancer dataset contain samples from patients with Liver Hepatocellular Carcinoma. It has three views: gene expression, miRNA expression and DNA Methylation. Based on clinical stages, patients are grouped into four categories: class one: stage I; class two: stage II; class three: IIIA, IIIB and IIIC; class four: stage IV.

#### TCGA.LUSC

Lung cancer dataset contain samples from patients with Lung Squamous Cell Carcinoma. It has three views: gene expression, miRNA expression and DNA Methylation. Based on clinical stages, patients are grouped into four categories: class one: stage IA, IB, IC; class two: stage II, IIA, IIB; class three: stage III; and class four: stage IV.

#### TCGA.SKCM

Melanoma cancer dataset contain samples from patients with skin cutaneous melanoma (SKCM). It has three views: gene expression, miRNA expression and DNA Methylation. Based on Clerk’s level in clinical data, patients are grouped into four categories: class one: Level I; class two: Level II; class three: Level III; and class four: Level IV.

#### TCGA.SARC

The Cancer Genome Atlas Sarcoma (TCGA.SARC) contain samples from patients suffering from sarcoma. It has three views: gene expression, miRNA expression and DNA Methylation. Based on the sample types in clinical data, patients are grouped into four categories.

#### TCGA.KIRC

The Cancer Genome Atlas Kidney Renal Clear Cell Carcinoma (TCGA-KIRC) contain samples from patients suffering from kidney cancer. It has three views: gene expression, miRNA expression and DNA Methylation. Based on clinical stages, patients are grouped into four categories: class one: stage I; class two: stage II; class three: stage III; and class four: stage IV.

#### TCGA.AML

TCGA.AML dataset contain samples from patients suffering from Acute Myeloid Leukemia. It has three views: gene expression, miRNA expression and DNA Methylation. Based on clinical data, patients are divided into four categories.

### Preprocessing of datasets

One of the common features of omics datasets is that the number of samples is much smaller than the number of features. Normalization of features in different omics is necessary for handling different distributions. Further, feature selection for dimensionality reduction is essential to provide different omics an equal prior opportunity to contribute to clustering. Dimensionality reduction is also crucial for keeping the most informative features, reducing the load on the clustering algorithm. In our approach, we have used an unsupervised feature selection technique, variance score. For this, we calculated the variance of each feature. Among them, top 22 − 24% features having highest scores are selected. The number of selected features for different benchmark datasets are given in Table 1.

### Evaluation metrics

To compare enAMOSA with other methods we have used two evaluation metrics, *normalized mutual information* (NMI) [47] and *adjusted rand index* (ARI) [48]. These metrics measure the similarity between the true and predicted partitions; higher values signify predicted class is more similar to true class.

## Results

### Input parameters

The proposed approach, *enAMOSA*, is based on the multiobjective optimization technique, AMOSA [35]. It has three main components: (i) initial temperature value (*T*_*max*_); (ii) cooling schedule; and (iii) number of iterations (*iter*) at each temperature.

The initial temperature is selected such that the algorithm can capture the entire search space. If initial temperature is set to too high, then it will accept all the proposed solutions and if set to too low it will transform into a greedy search. Here, the initial temperature (*T*_*max*_) is set to achieve an initial acceptance rate of approximately 50% on derogatory proposals. Here, *T*_*min*_ is set to 10^−3^. The initial temperature is selected based on the acceptance ratio of *ζ*, and average positive change in objective function, Δ*f*_*o*_ [49].

$$\begin{array}{c} T_{max}=-\frac{\Delta f_{o}}{\ln\zeta } \end{array}$$

Here *ζ* = 1/2,

$$\begin{array}{c} T_{max}=\frac{\Delta f_{o}}{\ln(2)} \end{array}$$

The cooling schedule determines the functional form of the change in temperature required in SA [35]. The temperature is changed using commonly used geometric schedule, *T*_*i* + 1_ = *α* × *T*_*i*_, where *α* is the cooling rate and 0 < *α* < 1. As stated in [35], value of *α* is chosen between 0.5 to 0.99. This cooling schedule is simple in nature. There is a need for a small number of transitions to be sufficient to reach the thermal equilibrium. Here, the value of *α* is set to 0.8, causing a sufficiently small number of transitions in temperature to reach equilibrium.

The number of iterations at each temperature is chosen so that the system is sufficiently close to the stationary distribution at that temperature [35]. Less number of iterations will significantly reduce the search space, and the solution will not reach the global optimal. For our problem, as the sample size is not considerably large, the iteration value is set to 100.

In Table 2, we have reported the parameter settings used in the experiments.

**Table 2:** Parameter settings for the proposed algorithm *enAMOSA*.

enAMOSA	
Max Temperature	100
Min Temperature	0.0001
# Iteration	100
Rate of cooling (*α*)	0.8
Soft Limit	40
Hard Limit	20

### Clustering performance

An extensive comparative study is performed to show the effectiveness of enAMOSA with respect to different approaches. The comparing approaches are briefly described below:

1. In order to show the effectiveness of using multiple clustering techniques to initialize the archive, we have developed different versions of enAMOSA clustering technique varying the base clustering algorithms. Abbreviations used *km*− K-means, *spec*− spectral, *cl*− complete linkage and *fs*− fast search. All these algorithms follow the exact steps of *enAMOSA*. Those are shown below:
  - enAMOSA_km_: this is the enAMOSA approach where only K-means clustering technique is used to generate the base partitionings. Here all the initial solutions of the archive are generated after running *K*-means clustering algorithm for different values of *K*. Other steps of this algorithm are very similar to those of *enAMOSA*.
  - enAMOSA_cl_: this is the enAMOSA approach where only complete linkage clustering technique is used to generate the base partitionings. Here all the initial solutions of the archive are generated after running complete linkage clustering technique for different values of *K*. Other steps of this algorithm are very similar to those of *enAMOSA*.
  - enAMOSA_fs_: this is the enAMOSA approach where only fast search clustering technique is used to generate the base partitionings. Here all the initial solutions of the archive are generated after running fast search clustering technique with different parameter values. Other steps of this algorithm are very similar to those of *enAMOSA*.
  - enAMOSA_spec_: this is the enAMOSA approach where only spectral clustering technique is used to generate the base partitionings. Here all the initial solutions of the archive are generated after running spectral clustering technique with different values of *K*. Other steps of this algorithm are very similar to those of *enAMOSA*.
  Further, as a part of our experimentation, we have also developed different versions of proposed *enAMOSA* approach where different combinations of size 2 / 3 base clustering algorithms are utilized for generating the initial solutions in the archive. Other steps of these approaches are very similar to those of *enAMOSA*. For Eg, *enAMOSA_km,spec_*, uses the K-means and spectral clustering for generating base partitionings and follows the exact steps of *enAMOSA*. The results of these different variants of *enAMOSA* are shown in Tables 3 and 4.
2. In order to show the efficacy of ensemble based perturbation operator in enAMOSA process, we have developed another ensemble based multiobjective multi-view based approach, namely, AMOSA(ensemble). The steps of this approach are enumerated below:
  - The initialization of the archive will be done similar to that of enAMOSA. Four different clustering techniques, *K*-means, complete linkage, spectral and fast search clustering are executed multiple times with varying parameter values and the number of clusters. The membership matrices generated by these clustering techniques are encoded in the form of solutions of the archive.
  - For the perturbation operator we have used the following operator. The simple binary mutation is applied on each membership matrix encoded as a string with some probability. The binary bit value is flipped with some probability. Some points are randomly selected and their membership values are changed.
  - In order to compute the objective functions, *V* number of membership matrices present in the string are obtained. The *conn-XB*-index values of all these *V* partitionings are calculated. The agreement index between these *V* partitionings is also calculated. The objective functions are $\left\{conn-XB_{1},conn-XB_{2},\ldots ,conn-XB_{V},\frac{1}{AI}\right\}$
  - AMOSA process is applied to simultaneously minimize these objective functions.
  Note that the above process is different from the proposed approach only in the use of perturbation operator. Unlike enAMOSA here normal binary perturbation operations are used to generate new solutions. Thus initial solutions generated by base clustering algorithms were not ensembled during the optimization process. Each individual solution is evolved separately without mixing with other solutions. This algorithm is developed to show that ensemble based mutation operation indeed plays an important role in generating good solutions.
3. In order to show the potency of multiobjective based multiview clustering, we have also compared the performance of enAMOSA with state-of-the art multiview based classification techniques, namely MVDA (unsupervised) algorithm [16], LRAcluster [18], PINS [21], SNF [23] and iClusterBayes [28].

**Table 3:** Comparison of Normalized Mutual Information (NMI) scores of different combinations of our proposed approach.

	BRC	BRC.1	BRC.2	MSKCC	GBM	OVG	COAD	LIHC	LUSC	SKCM	SARC	KIRC	AML
**enAMOSA_km,spec_**	0.4461	0.4714	0.3842	0.1495	0.4816	0.1194	0.1377	0.1209	0.3435	0.0814	0.09708	0.0910	0.4615
**enAMOSA_km,cl_**	0.4131	0.4219	0.4692	0.0979	0.4401	0.1107	0.1201	0.0984	0.3321	0.0618	0.0483	0.0291	0.3078
**enAMOSA_km,fs_**	0.4601	0.4679	0.4097	0.1401	0.4487	0.1303	0.1417	0.1334	0.3647	0.0796	0.1040	0.0951	0.5157
**enAMOSA_cl,spec_**	0.4515	0.4653	0.3904	0.1425	0.4803	0.1147	0.1207	0.1134	0.3476	0.0784	0.0736	0.0891	0.4574
**enAMOSA_cl,fs_**	0.4641	0.4730	0.4012	0.1498	0.4817	0.1203	0.1297	0.1219	0.3574	0.07807	0.1022	0.01074	0.5098
**enAMOSA_spec,fs_**	0.4689	0.4717	0.4397	0.1521	0.4927	0.1104	0.1514	0.1298	0.3651	0.1126	0.1013	0.1095	0.5231
**enAMOSA_km,cl,fs_**	0.4787	0.5105	0.4475	0.1735	0.5094	0.1394	0.1704	0.1473	0.3747	0.1319	0.0985	0.1025	0.5201
**enAMOSA_cl,fs,spec_**	0.4702	0.5447	0.4778	0.1916	0.5407	0.2236	0.2012	0.1603	0.4096	0.1594	0.1102	0.1154	0.5487
**enAMOSA_cl,spec,km_**	0.4707	0.4804	0.4584	0.1605	0.5146	0.1264	0.1537	0.13199	0.3815	0.1409	0.1017	0.0920	0.4701
**enAMOSA_km,spec,fs_**	0.4772	0.5546	0.4760	0.2066	0.5419	0.2176	0.1952	0.1693	0.4106	0.1609	0.1130	0.1161	0.5507

**Table 4:** Comparison of Adjusted Rand Index (ARI) scores of different combinations of our proposed approach.

	BRC	BRC.1	BRC.2	MSKCC	GBM	OVG	COAD	LIHC	LUSC	SKCM	SARC	KIRC	AML
**enAMOSA_km,spec_**	0.4007	0.3717	0.3212	0.0998	0.3921	0.1091	0.02851	0.0163	0.3164	0.03205	0.0987	0.1048	0.4015
**enAMOSA_km,cl_**	0.3681	0.3402	0.2954	0.0481	0.3620	0.0493	0.0224	0.0098	0.1712	0.0216	0.0190	0.0401	0.3634
**enAMOSA_km,fs_**	0.4116	0.3797	0.3207	0.1034	0.3975	0.1017	0.03196	0.0185	0.2705	0.0330	0.1045	0.1314	0.4184
**enAMOSA_cl,spec_**	0.3918	0.3697	0.3102	0.0981	0.3816	0.1034	0.02961	0.0185	0.2624	0.03105	0.0991	0.1034	0.4087
**enAMOSA_cl,fs_**	0.4066	0.3757	0.3198	0.1018	0.3943	0.1009	0.03114	0.0179	0.2695	0.0321	0.1051	0.1326	0.4161
**enAMOSA_spec,fs_**	0.4216	0.4375	0.3792	0.1085	0.4369	0.1078	0.10184	0.0704	0.2459	0.0253	0.1098	0.1311	0.4201
**enAMOSA_km,cl,fs_**	0.4291	0.5120	0.3915	0.1294	0.4593	0.1011	0.1211	0.0935	0.2901	0.0843	0.1057	0.1319	0.4198
**enAMOSA_cl,fs,spec_**	0.4501	0.5284	0.4201	0.1513	0.4710	0.1012	0.1213	0.1016	0.3103	0.1023	0.1107	0.1402	0.4215
**enAMOSA_cl,spec,km_**	0.4393	0.5101	0.40914	0.1302	0.4601	0.1008	0.1461	0.1015	0.3091	0.0879	0.1008	0.1084	0.4087
**enAMOSA_km,spec,fs_**	0.4513	0.5304	0.4284	0.1601	0.4709	0.094	0.1437	0.1065	0.3002	0.0934	0.1103	0.1412	0.4208

**Fig 4:**
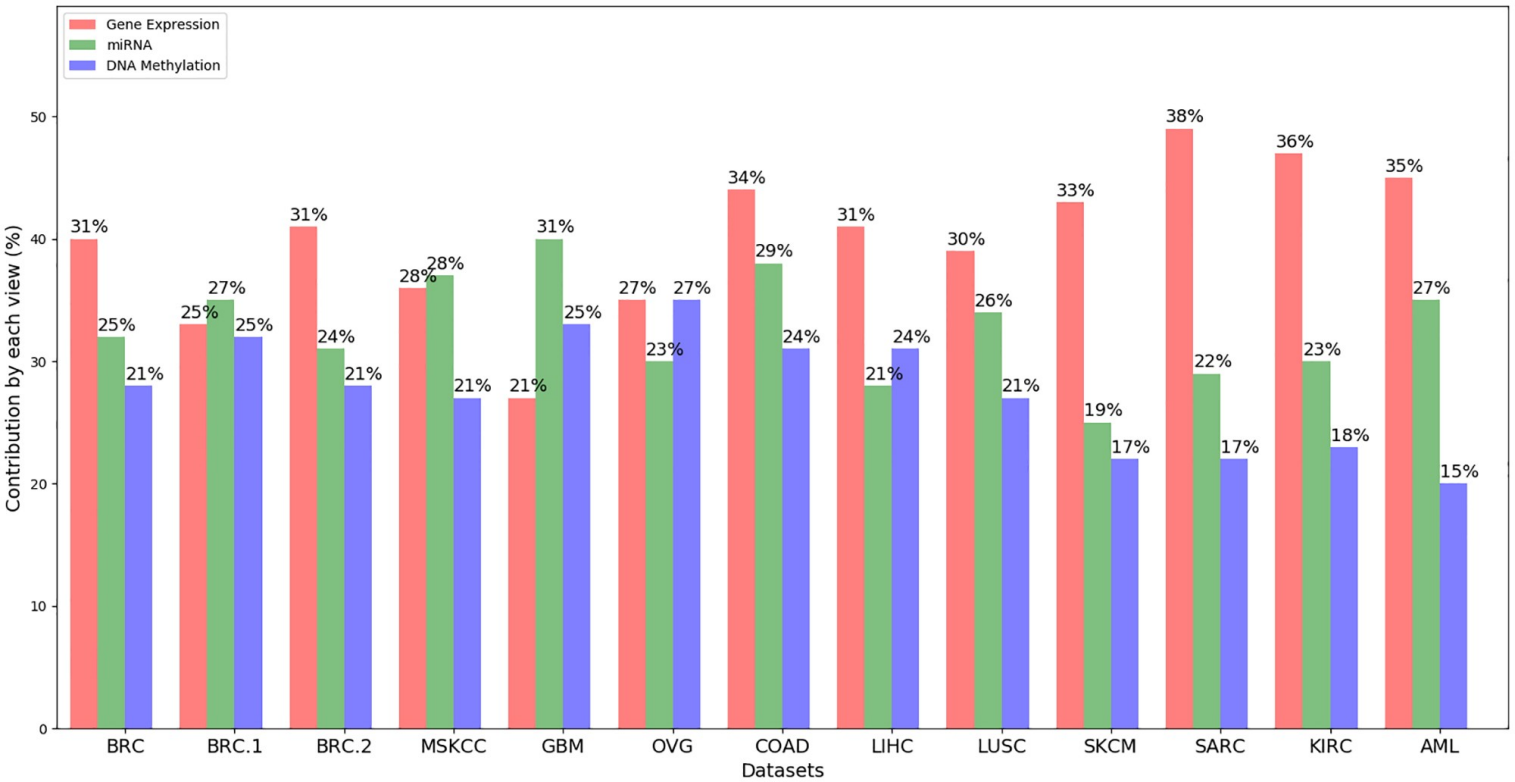
Contribution by each view in clustering.

Using Eq 12, we have calculated the degree of contribution by each view in the final clustering obtained. Contributions computed for different views are shown in Fig 4.

$$\begin{array}{c} Degree_{v}=\frac{∥Adj^{v}\cap Adj^{\cup v}∥}{∥Adj^{\cup v}∥} \end{array}$$ (12)

Here, *Adj*^*v*^ = the adjoint matrix of the partitioning obtained using view *v Adj*^∪*v*^ = the adjoint matrix corresponding to the final consensus partitioning.

### Statistical significance test

For statistical significance test we have used a non-parametric test one-way Analysis of Variance (ANOVA) because it is independent of the distribution type of the dataset. The test is performed at 1% significance level. Results obtained by all the seven algorithms for each dataset are divided into seven groups. One-way ANOVA is conducted between enAMOSA group and remaining groups and results are reported in Table 5. All the p-values reported in Table 5 are less than 0.01. These values establish that improvements obtained by enAMOSA over other comparing algorithms are statistically significant.

**Table 5:** The p-values reported by one-way ANOVA test on comparing *enAMOSA* with other methods.

	BRC	BRC.1	BRC.2	MSKCC	GBM	OVG	COAD	LIHC	LUSC	SKCM	SARC	KIRC	AML
**enAMOSA_km_**	9.35e-36	1.38e-35	2.84e-44	8.46e-51	1.01e-31	1.61e-17	7.77e-4	5.65e-5	6.20e-10	9.05e-8	1.28e-5	3.56e-4	8.07e-5
**enAMOSA_cl_**	3.94e-63	1.18e-54	7.69e-50	8.18e-65	3.28e-57	3.11e-22	5.32e-9	2.31e-7	3.67e-7	7.11e-10	2.01e-4	2.01e-7	3.17e-5
**enAMOSA_spec_**	3.55e-15	3.08e-18	4.00e-20	1.93e-14	1.98e-28	1.79e-24	1.23e-8	2.14e-5	1.05e-11	8.12e-6	5.18e-5	2.28e-7	1.21e-4
**enAMOSA_fs_**	1.28e-31	2.56e-30	7.07e-32	3.65e-41	6.22e-42	7.05e-25	3.24e-14	1.27e-9	5.42e-8	79.12e-7	6.65e-5	7.22e-4	6.05e-5
**AMOSA (ensemble)**	7.24e-25	5.05e-28	2.49e-31	1.78e-27	3.14e-33	2.82e-26	2.17e-24	3.24e-7	2.78e-8	5.31e-7	0.44e-7	5.12e-7	4.64e-6
**LRAcluster**	8.94e-28	3.11e-28	4.77e-26	7.84e-28	4.11e-28	2.57e-26	1.84e-13	3.76e-11	1.11e-4	1.61e-7	3.74e-8	4.41e-4	4.37e-5
**PINS**	1.44e-7	1.12e-7	8.64e-6	2.73e-7	8.93e-9	4.53e-5	2.44e-9	6.72e-6	6.50e-7	1.63e-4	3.08e-8	1.00e-4	1.93e-4
**SNF**	1.26e-12	5.71e-4	8.63e-8	7.64e-9	2.77e-5	6.23e-7	1.19e-7	6.34e-12	2.63e-10	6.22e-11	8.94e-9	7.53e-11	1.43e-9
**iClusterBayes**	0.0045	0.0118	0.0086	0	0.0357	0.0023	0.0076	0.0048	0.0034	0.0043	4.93e-9	4.51e-5	2.24e-10
**MVDA**	3.74e-12	1.41e-4	7.37e-7	1.24e-15	4.13e-6	8.73e-10	9.87e-11	1.45e-17	1.35e-5	7.86e-7	4.11e-8	2.57e-6	1.84e-3

### Gene marker identification

From the clustering results obtained by enAMOSA on the OXF.BRC.1 dataset, we tried to extract the group of genes which have mainly contributed in patient classification. There are four patient classes in OXF.BRC.1 data set, viz., Her2, Basal, LumA, LumB. To identify the gene markers from Her2 class, we solved a binary classification problem. Two groups are created, one containing the samples from Her2 class and the other containing samples from rest of the classes. After considering both the groups, Signal-to-Noise Ratio (SNR) [50] is calculated for each of the genes. It is defined as,

$$\begin{array}{c} SNR=\frac{\mu_{1}-\mu_{2}}{\sigma_{1}+\sigma_{2}}\times 100, \end{array}$$ (13)

where *σ*_*j*_ and *μ*_*j*_, *j* ∈ [1, 2], respectively, denote the standard deviation and the mean of class *j* for the corresponding gene. Higher SNR value for an individual gene signifies that it is having higher expression value for the class it belongs to and lower expression values for others. Finally total 10 genes are selected from the SNR list, with top 5 genes having highest SNR values (up regulated genes) and bottom 5 genes having lowest SNR values (down regulated genes). Similarly like Her2, the process is repeated for other classes too present in the dataset.

#### Gene markers for OXF.BRC.1 dataset

**Fig 5:**
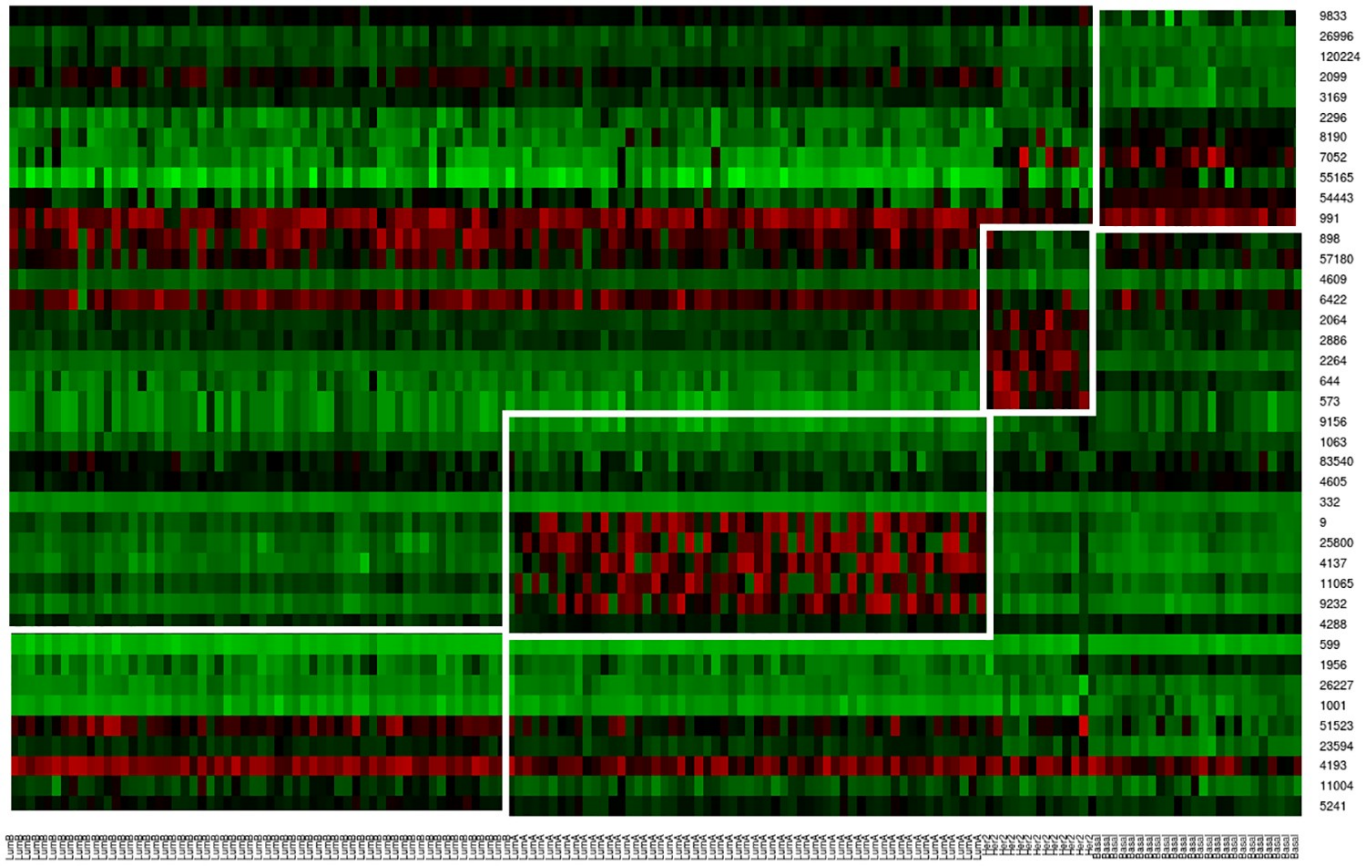
Heatmap to show the expression levels of the selected gene markers for each subclass in OXF.BRC.1 dataset.

OXF.BRC.1 dataset is having 4 classes, so a total of 40 genes are obtained with 20 up regulated genes and 20 down regulated genes. Fig 5 shows the heatmap plot of these genes along with their class names on X-axis. Here red signifies higher expression levels, green signifies lower expression levels and black signifies moderate expression levels. It is also seen from the Fig 5 that for a particular tumor class identified genes are either up-regulated or down-regulated. List of selected gene markers for Her2, Basal, LumA, LumB classes are reported in Table 6. Note that a gene up-regulated in one class can be down-regulated in another.

**Table 6:** Selected 10 gene markers for OXF.BRC.1 dataset.

Her2		Basal		LumA		LumB	
Gene ID	Down/Up	Gene ID	Down/Up	Gene ID	Down/Up	Gene ID	Down/Up
2064	**Down**	2296	**Down**	9	**Down**	51523	**Down**
2886	**Down**	8190	**Down**	25800	**Down**	23594	**Down**
2264	**Down**	7052	**Down**	4137	**Down**	4193	**Down**
644	**Down**	55165	**Down**	11065	**Down**	11004	**Down**
573	**Down**	54443	**Down**	9232	**Down**	5241	**Down**
991	**Up**	9833	**Up**	9156	**Up**	4288	**Up**
898	**Up**	26996	**Up**	1063	**Up**	599	**Up**
57180	**Up**	120224	**Up**	83540	**Up**	1956	**Up**
4609	**Up**	2099	**Up**	4605	**Up**	26227	**Up**
6422	**Up**	3169	**Up**	332	**Up**	1001	**Up**

### Biological significance test

To show the biological significance of selected genes, a biological significance test is conducted using Gene ontology consortium (http://www.geneontology.org/). For each GO term, the percentage of genes sharing that term among the genes of that cluster (% Cluster) and among the whole genome (%Genome) has been reported in Table 7. From the results, it can be seen that the genes belonging to the same cluster share a higher percentage of GO terms compared to the whole genome. This signifies that the genes of a particular cluster are more involved in the similar biological process compared to the remaining genes of the genome.

**Table 7:** Significant shared Gene Ontology (GO) terms by gene markers.

Classes	Gene Ontology(GO) term	(%) Genome	(%)Cluster
**Her2**	regulation of catalytic activity: GO:0050790	47%	50%
	regulation of cell proliferation: GO:0042127	31%	40%
	negative regulation of programmed cell death: GO:0043069	50%	50%
	negative regulation of apoptotic process: GO:0043066	2%	20%
	positive regulation of cell proliferation: GO:0008284	3%	40%
	negative regulation of cell death: GO:0060548	5%	38%
**Basal**	biological process: GO:0008150	50%	50%
	biological regulation: GO:0065007	52%	60%
	signal transduction: GO:0007165	6%	10%
	nitrogen compound metabolic process: GO:0006807	27%	30%
	multicellular organismal process: GO:0032501	3%	30%
**LumA**	cell cycle: GO:0007049	30%	46%
	regulation of chromosome organization: GO:0033044	2%	20%
	organelle fission: GO:0048285	3%	10%
	regulation of chromosome segregation: GO:0051983	8%	10%
	mitotic nuclear division: GO:0140014	3%	20%
**LumB**	regulation of biological process: GO:0050789	17%	20%
	regulation of cellular process: GO:0050794	0.5%	10%
	multicellular organism development: GO:0007275 GO:0007275	1.3%	10%
	regulation of macromolecule metabolic process: GO:0060255	16%	20%
	organic substance biosynthetic process: GO:1901576	21%	30%

## Discussion

The average NMI and ARI values obtained by the execution (20 times) of our proposed method, enAMOSA, on all the 13 datasets are shown in Tables 8 and 9, respectively. From the Tables 8 and 9, it is observed that the results obtained by our proposed methodology outperform the results obtained by other state-of-the-art single objective algorithms (MVDA (unsupervised) algorithm [16], LRAcluster [18], PINS [21], SNF [23] and iClusterBayes [28]) by 10 − 11% (approx.) and 10 − 14% (approx.) in terms of NMI and ARI, respectively. Comparison of *enAMOSA* with other baseline versions (enAMOSA_km_, enAMOSA_spec_, enAMOSA_cl_ and enAMOSA_fs_) shows that combination of all the four base partitions (i.e., *enAMOSA*) performs better than its single base partition counterparts (enAMOSA_km_, enAMOSA_spec_, enAMOSA_cl_ and enAMOSA_fs_) by 5 − 10% (approx.) and 8 − 11%(approx.) in terms of NMI and ARI, respectively. To show the effectiveness of the new perturbation operator, *enAMOSA* is compared with *AMOSA(ensemble)*. NMI and ARI scores obtained by *enAMOSA* exceed *AMOSA(ensemble)* by 5 − 9%(approx.) and 8 − 10% (approx.), respectively. Results reflect the efficiency of the proposed integrated approach of ensemble and multi-objective algorithm through new perturbation operator over using them separately.

**Table 8:** Comparison of Normalized Mutual Information (NMI) scores of our proposed approach (*enAMOSA*) with other baseline approaches and state-of-the art methods.

	BRC	BRC.1	BRC.2	MSKCC	GBM	OVG	COAD	LIHC	LUSC	SKCM	SARC	KIRC	AML
**enAMOSA**	**0.5092**	**0.5714**	**0.4980**	**0.2296**	**0.5649**	**0.2347**	**0.2151**	**0.1894**	**0.4216**	**0.1809**	**0.1147**	**0.1208**	**0.5607**
**enAMOSA_km_**	0.4021	0.4185	0.3618	0.0906	0.4383	0.0797	0.0971	0.0594	0.3021	0.0281	0.0463	0.0184	0.3017
**enAMOSA_cl_**	0.4067	0.4206	0.3531	0.1076	0.4354	0.0814	0.1014	0.0498	0.2841	0.0212	0.0315	0.0281	0.2962
**enAMOSA_spec_**	0.4215	0.4418	0.3615	0.1206	0.4519	0.1067	0.1098	0.0961	0.3196	0.0684	0.0748	0.0907	0.4517
**enAMOSA_fs_**	0.4375	0.4668	0.3798	0.1179	0.4708	0.1201	0.1147	0.0948	0.3089	0.0716	0.1005	0.1104	0.5141
**AMOSA (ensemble)**	0.4581	0.5071	0.4104	0.1390	0.4615	0.1421	0.1227	0.1103	0.3284	0.1097	0.1091	0.1109	0.5207
**LRAcluster**	0.0146	0.0232	0.0118	0.1098	0.0532	0.0304	0.0328	0.0573	0.0672	0.0483	0.0475	0.0389	0.3629
**PINS**	0.0118	0.0146	0.00392	0.0572	0.0153	0.0095	0.0459	0.0348	0.0237	0.0382	0.0262	0.0279	0.2219
**SNF**	0.0358	0.0475	0.0153	0.0098	0.0026	0.0068	0.0332	0.0129	0.0082	0.0088	0.0233	0.0908	0.4349
**iClusterBayes**	0.0121	0.09931	0.0153	0.0780	0.0306	0.0081	0.0106	0.0258	0.0112	0.0044	0.0177	0.0108	0.0894
**MVDA**	0.3912	0.4034	0.3403	0.1124	0.4213	0.0863	0.0793	0.0175	0.2594	0.0195	0.0321	0.0655	0.2871

**Table 9:** Comparison of Adjusted Rand Index (ARI) scores of our proposed approach (*enAMOSA*) with other baseline methods and state-of-the art methods.

	BRC	BRC.1	BRC.2	MSKCC	GBM	OVG	COAD	LIHC	LUSC	SKCM	SARC	KIRC	AML
**enAMOSA**	**0.4723**	**0.5534**	**0.4414**	**0.1721**	**0.4809**	**0.1157**	**0.1461**	**0.1215**	**0.3471**	**0.1105**	**0.1215**	**0.1537**	**0.4315**
**enAMOSA_km_**	0.3641	0.3471	0.2815	0.0448	0.3561	0.0430	0.02051	0.0083	0.1573	0.0161	0.0189	0.0384	0.3412
**enAMOSA_cl_**	0.3651	0.3384	0.2901	0.0457	0.3620	0.0473	0.02110	0.0082	0.1615	0.0186	0.0175	0.0115	0.3603
**enAMOSA_spec_**	0.3805	0.3547	0.2981	0.0935	0.3726	0.0984	0.02751	0.0096	0.2136	0.0228	0.0984	0.1045	0.3942
**enAMOSA_fs_**	0.3916	0.3675	0.3102	0.0958	0.3864	0.0953	0.02814	0.0094	0.2219	0.0231	0.1041	0.1308	0.4107
**AMOSA (ensemble)**	0.3943	0.3861	0.3306	0.1006	0.3937	0.1002	0.0447	0.0108	00.2419	0.0259	0.1114	0.1137	0.4208
**LRAcluster**	0.0086	0.0112	0.0012	0.0105	0.0076	0.0051	0.0184	0.0054	0.0098	0.0055	0.0263	0.0392	0.2546
**PINS**	0.0144	0.0112	0.00864	0.01142	0.0089	0.0045	0.0244	0.0067	0.0065	0.0016	0.0152	0.0136	0.1195
**SNF**	0.0126	0.0057	0.0863	0.00081	0.0027	0.0062	0.0119	0.0063	0.0026	0.00062	0.0238	0.0157	0.3667
**iClusterBayes**	0.0045	0.0118	0.0086	0.0171	0.0357	0.0023	0.0076	0.0048	0.0034	0.0043	0.0399	0.0288	0.0482
**MVDA**	0.2457	0.3441	0.2831	0.067	0.3441	0.0614	0.0194	0.0085	0.1351	0.0145	0.02366	0.0355	0.2021

To further explore the importance of diversity in the base partitions, all possible combinations of the base partitions are generated and the results of NMI and ARI are presented in Tables 3 and 4 respectively. Comparing these results with that obtained by the proposed algorithm *enAMOSA* (from Tables 8 and 9) it is observed that the proposed method outperforms its counterparts by 2 − 4% (approx.) for both NMI and ARI. The following observations are drawn from careful analysis of the results in Tables 3 and 4:

1. The hypothesis of the work is that the diversity in the initial solutions will allow the algorithm to capture more accurate cluster structures. From Tables 3 and 4, we can see that the NMI and ARI values obtained by combined base partitions are higher compared to their single counterparts in Tables 8 and 9 respectively. Further, within Tables 3 and 4 it is seen that combination of 3 base partitions produces higher results compared to the combination of 2 base partitions. For example, results obtained by *enAMOSA_cl,km,spec_* are better compared to any of its 2 base partitions like *enAMOSA_cl,km_*, *enAMOSA_cl,spec_* and *enAMOSA_km,spec_*. Similar results are true for other combinations also that are reported in Tables 3 and 4. Results support the initial hypothesis of the work.
2. The performance of the algorithm depends on the type of base partitions used for populating the archive initially. In the worst case scenario, *enAMOSA* generates results comparable to the best base partition solution the archive is initialized with. By this we mean that, suppose initially the archive is initialized with solutions obtained from two different base algorithms, one producing good results and other producing bad. Final results obtained from *enAMOSA* will be comparable to that of the good results. For example, for dataset *SARC* in Table 8, we can see *enAMOSA_km_* and *enAMOSA_fs_* provide NMI values of 0.0184 and 0.1005, respectively. When the partitions obtained from base algorithms Kmeans and fast search are used jointly, the NMI result obtained by *enAMOSA_km,fs_* on *SARC* dataset is 0.1040, reported in Table 3. By analyzing Tables 3 and 4, it is observed that similar pattern is followed for other datasets also. At least, *enAMOSA* ensures to generate best possible solution among the base partitions.
3. From Tables 3 and 4, it is also observed that enAMOSA_km,spec,fs_ performs better than other algorithms presented in these tables for most of the datasets. A closer analysis shows that mainly density-based clustering algorithms (fast search and spectral clustering) capture better structures from the datasets compared to K-means and hierarchical. It may be because density-based clustering algorithms are capable of capturing arbitrary cluster structures from the datasets.

Apart from NMI and ARI scores, we have also reported the F1-measure and accuracy score obtained by *enAMOSA* on all the 13 benchmark datasets in the Table 10.

**Table 10:** F1-measure and accuracy values obtained by *enAMOSA* for all the datasets.

Datasets	F1-measure	Accuracy
TCGA.BRC	0.6592	0.6814
OXF.BRC.1	0.7048	0.7184
OXF.BRC.2	0.6067	0.6126
MSKCC	0.5146	0.5541
TCGA.GBM	0.6975	0.7068
TCGA.OVG	0.5012	0.5236
TCGA.COAD	0.4905	0.5131
TCGA.LIHC	0.4718	0.4946
TCGA.LUSC	0.6056	0.6479
TCGA.SKCM	0.4861	0.4931
TCGA.SARC	0.4315	0.4415
TCGA.KIRC	0.4174	0.4212
TCGA.AML	0.6904	0.6882

**Fig 6:**
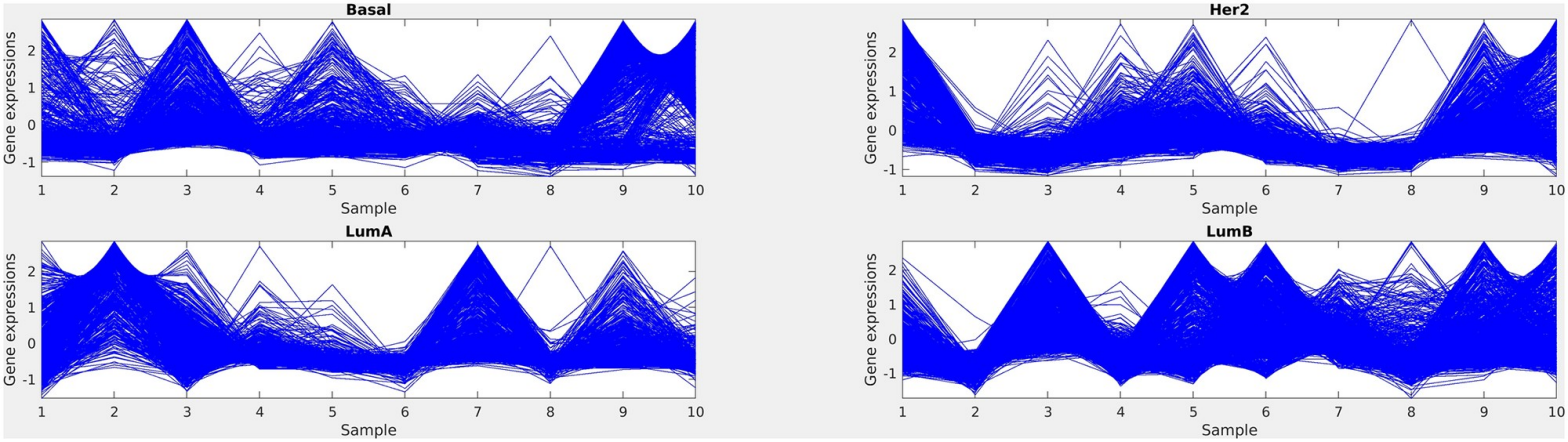
Gene expression profile plot for each subclass in OXF.BRC.1 dataset.

In Fig 6, we have reported the gene expression profile plot for each individual classes (Basal, Her2, LumA and LumB) of OXF.BRC.1 dataset. The compactness of the structures shows that the clustered samples share the same type of gene expressions, i.e, within a cluster genes have good coherence among them.

In Table 11, we have reported the execution time (in seconds) for all the algorithms used in the experiment. All the algorithms are executed on machine having intel Core i5 7th Generation processor with 8GB of RAM. The time is calculated by taking the average over 20 runs of the algorithms. Execution time of *enAMOSA* is comparable to that of *LRAcluster* and *iClusterBayes*.

**Table 11:** Execution time of the algorithms in seconds.

	BRC	BRC.1	BRC.2	MSKCC	GBM	OVG	COAD	LIHC	LUSC	SKCM	SARC	KIRC	AML
**enAMOSA**	9531.21	4389.12	4380.98	727.08	2045.80	4419.02	2241.11	4373.24	4352.51	6533.02	3016.14	2045.03	978.42
**LRAcluster**	15034.51	3421.16	3415.77	972.30	2055.93	3417.27	2104.53	5918.45	5074.86	8550.49	3057.00	1569.91	1025.22
**PINS**	1219.78	612.71	614.45	121.45	356.52	634.88	379.28	371.29	596.02	798.99	376.72	379.28	206.98
**SNF**	65.71	19.61	20.71	12.04	13.72	20.07	15.89	27.21	24.31	36.23	17.30	15.89	14.90
**iClusterBayes**	9041.95	4503.10	4523.06	1014.06	2923.39	4543.08	3635.03	5669.00	5309.83	6776.40	4197.39	3635.03	1847.40
**MVDA**	222.11	146.73	140.27	153.54	190.24	136.37	197.62	224.15	141.09	214.08	175.583	211.07	225.11

### Scalability analysis

An important aspect of performance analysis is the study of how algorithm performance varies with parameters. In particular, we may evaluate the scalability of the algorithm, that is, how effectively it can use an increased number of samples. From the time complexity equation, Eq 6, it is observed that the execution time of the model depends on the total number of iterations (*TotalIter*), number of samples (*n*), number of clusters (*r*), size of archive (*N*) and number of objective functions (*M*). Now, the total number of iteration (*TotalIter*), size of archive (*N*) and number of objective functions (*M*) are fixed for all the datasets. As for the number of clusters are concerned, in our algorithm, the number of clusters is not fixed for any particular dataset, the algorithm automatically determines the number of clusters. So, the increase in execution time depends on the number of samples (*n*) present in the datasets. By analyzing the numeric values obtained by empirical studies in Table 11, clearly supports our finding. *TCGA.BRC* has the highest number of samples (629), it has the highest execution time of 9631.21 seconds and *MSKCC.PRA* has the lowest number of samples (151), it has the lowest execution time of 727.08 seconds. Similar kind of results are seen for other datasets also in Table 11, that is, execution time increases with an increase in sample points. The results reported in Table 11 reveals that the execution time of the algorithm is not huge with the increase in the number of samples in the data set; it converges in polynomial time even with a large number of samples and also the execution time is comparable to the state-of-the-art method *iClusterBayes*.

## Conclusion

In order to properly subclassify the patient data, consideration of multiple views is highly solicited. A single clustering method is not enough to capture all possible structures in a dataset. This is a multi-view classification problem which is solved with the help of the proposed multiobjective based multi-view cluster ensemble based technique.

In the current paper, we have proposed a multiobjective based cluster ensemble technique for multi-view classification. Initially different simple clustering algorithms are applied to generate some base partitionings by varying the number of clusters. These initial solutions are finally combined using some cluster ensemble based operators. The goodness of the individual partitionings obtained using different views is measured using a connectivity-based internal cluster validity index, namely *conn-XB* and an agreement index computing the agreement amongst the partitionings captured on different views. The values of these measures are simultaneously optimized using the search capability of AMOSA, which is a multiobjective simulated annealing based optimization technique. Obtained results on 13 cancer data sets illustrate the utility of the proposed approach for patient sub-classification task. An extensive comparative study has been conducted to show the efficacy of individual components of the proposed enAMOSA approach. Some approaches are developed to show the utility of initialization step of enAMOSA; further another multi-view based cluster ensemble technique is developed which utilizes some normal mutation operators instead of using an ensemble-based operator. This comparative study reveals that all the components of the proposed approach, enAMOSA are important.

Some of the important findings we made are (i) proposed algorithm successfully captures complex heterogeneous structures from multi-omics data compared to other state-of-the-art methodology; (ii) the proposed perturbation operator proves effective in integrating the ensemble technique with multi-objective technique. The comparative results support its effectiveness; (iii) the algorithm, *enAMOSA*, effectively combines multiple views having different number of clusters; (iv) the execution time of the algorithm is not huge; it converges in polynomial time and also the execution time is comparable to the state-of-the-art method *iClusterBayes*. Study of the various comparative results presented in this paper supports our findings.

In future research, works will be carried out in developing a multi-view based biclustering framework. The developed multi-view based clustering techniques will be applied for solving some real-life problems of social media data. Documents can also be represented using multiple views. Thus many of the document classification problems can be solved using the developed multiobjective based multi-view clustering technique.
